# Building an analytical framework for tobacco-related information on social media: an exploratory analysis with generative AI assistance

**DOI:** 10.1186/s12889-025-24767-w

**Published:** 2025-10-28

**Authors:** Eileen Han, Miao Feng, Pamela Ling

**Affiliations:** 1 Center for Tobacco Control Research and Education, University of California, 530 Parnassus Ave., San Francisco, CA 94143 USA; 2 Rice University, 6100 South Main St, Houston, TX 77005 USA; 3 Department of Medicine, Division of General Internal Medicine, University of California, San Francisco, CA 94143 USA; 4 Social Data Collaboratory, NORC at the University of Chicago, Chicago, IL 60603 USA

**Keywords:** E-cigarettes, Misleading information, Tobacco control, Social media, Twitter

## Abstract

**Background:**

The propagation of tobacco-related information that is inconsistent with public health guide significantly impacts public health, particularly affecting people with less access to reliable information sources (such as those with lower education), who may also suffer disproportionate tobacco-related morbidity and mortality. This study develops a multi-dimensional analytical framework for identifying and categorizing tobacco-related information on social media. Using a dataset of tweets, the framework was constructed through qualitative analysis, which was then compared with an exploratory, AI-assisted analysis to assess the capabilities of current automated tools.

**Methods:**

A collection of 3.4 million tweets related to tobacco and nicotine was refined to 842,754 after removing irrelevant and duplicate posts. LDA topic modeling identified six unique topics, from which two randomly selected samples of tweets were drawn to perform qualitative analysis and AI-assisted analysis to identify categories of tobacco information.

**Results:**

The identified tobacco-related information was categorized by three dimensions (1) content, including safety and health effects, cessation, substance, and policy; (2) type of falsehood, which included fabrication and unsubstantiated claims, misrepresentations, and distortions; and (3) source, ranging from individuals and retail stores to advocacy groups and influencers. A notable finding was the prevalence of policy-related discussions of tobacco information on Twitter (X), highlighting this often-overlooked domain. The controversy over vaping has amplified pro-vaping voices on social media, with content frequently misinterpreting scientific findings, policies, and expert opinions, reflecting more nuanced and difficult to recognize falsehood in the misleading content.

**Conclusion:**

This study offers a comprehensive framework for analyzing tobacco-related information on social media, emphasizing key issues in policy debates and the presence of conspiracy narratives. This framework can inform the design of interventions for less informed populations and enhance data annotation for machine learning tasks.

## Background

Tobacco-related misinformation on social media can significantly impact public health by shaping false perceptions and beliefs. Misinformation may mislead users of tobacco products in decision-making and potentially harms the health of communities most impacted by tobacco product use and its health consequences, contributing to health disparities [1]. The potential impact of misleading information is greater for groups with higher smoking rates, such as those with lower income or education, who may also have less access to reliable information [2]. For example, misperceptions about the risks and benefits of e-cigarettes could affect decisions to switch or quit [3]. With the tobacco industry’s continuing targeted marketing, inconclusive data on the risks and benefits of emerging novel products, and inequalities in accessing reliable and quality information, improving information literacy could mitigate disparities in tobacco-related health outcomes [4].

While health misinformation studies are abundant, as smoking and drug-related misinformation is prevalent on X (previously and herein: Twitter) [5], relatively few studies directly address tobacco-related misinformation on social media. Understanding the common characteristics of tobacco misinformation on social media could help public health researchers and experts design interventions for targeted populations. However, as new tobacco products rapidly emerge, misinformation related to these products also grows quickly, with potentially misleading marketing messages and social media discussions [1]. It is challenging for evidence-based research and public health surveillance systems to keep up and address the potential risks of new and evolving products [6]. A framework that captures common patterns and characteristics of existing tobacco information and that can adapt to emerging trends can inform our understanding of messages and strategies to correct misunderstanding.

Previous studies on tobacco misinformation have focused on single issues, such as misunderstanding of nicotine, the health risks and benefits of e-cigarettes, or the relationship between vaping and COVID-19 [7–10]. Some studies have tested selected message effects [11, 12] and corrective messages [13]. However, to our knowledge, no study has provided a way to systematically organize tobacco-related information on social media. In the area of vaccines, researchers have developed typologies or taxonomies about anti-vaccine misinformation based on online content and design features [14, 15]. Therefore, the primary aim of this study is to develop a systematic framework for organizing tobacco-related misinformation on social media. To achieve this, we combined in-depth qualitative analysis by human researchers with an exploratory use of generative AI, allowing us to build the framework while simultaneously evaluating the strengths and limitations of automated tools for this complex task. the.

## Methods

### Data collection rationale

The tobacco industry has been identified as a source of misinformation through misleading public relation posts on social media [16, 17]. Studies also show that while the tobacco industry’s social media accounts may seem compliant with marketing restrictions, such as setting age limits for content viewing and refraining from using words indicating health benefits, industry messages can share hashtags with public tweets produced by others about the same topic without such restrictions [18]. Therefore, tobacco industry-produced content serves as a reasonable starting point for exploring the use of similar hashtags and keywords in a broader context. In this study, we started with an analysis of industry tweets to identify priority topics in recent years and used their top-prioritized hashtags and corresponding keywords (hashtags) to further inform our search for general public tweets. All tweets from two leading transnational tobacco companies, British American Tobacco and Philip Morris International, were collected using the Twitter application programming interface (API) from January 2020 to December 2022.

**Fig. 1:**
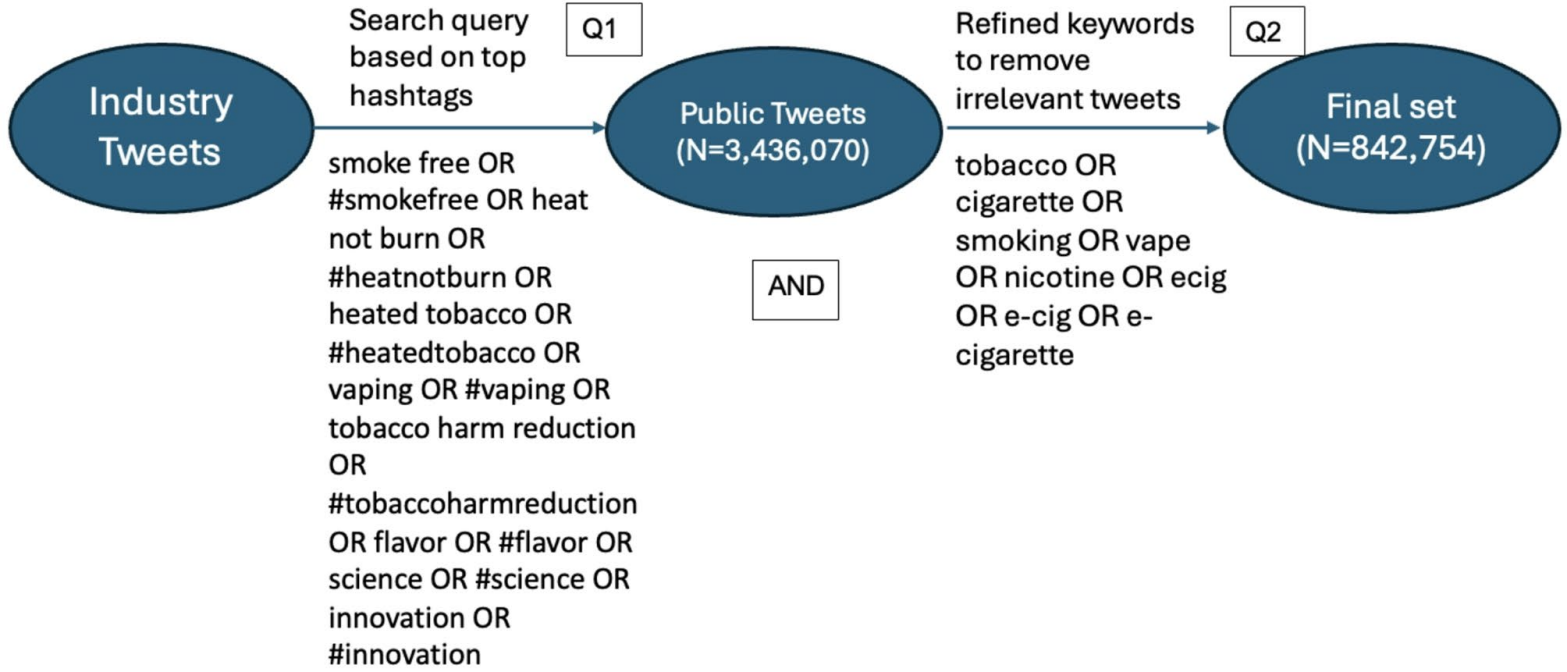
Data collection and processing

Figure 1 shows the development process for search queries. The top 10 hashtags the two tobacco companies used between 2020 and 2022 were identified in the dataset of industry tweets, and company-specific-hashtags (with company names or slogans in them) were excluded in order to capture a broader public conversation on these topics, rather than limiting our search to tweets directly engaging with corporate marketing. Eight hashtags remained in the search query, and together with their corresponding keywords (Q1), they were used to search tweets in a larger database that collected public tobacco-related posts since 2015 using Twitter’s streaming API [19] (Fig. 1). The search resulted in a total of 3,436,070 tweets dated from January 1, 2020, to December 31, 2022 (Public Tweets). Because these search keywords included hashtags used by tobacco companies are widely used in other contexts (e.g. #science and #innovation), the resulting public tweets contained many thematically irrelevant tweets. Therefore, an additional search of the public tweets was conducted with a few tobacco-related key terms that did not appear in the initial search but were commonly used in tobacco-related tweets (Q2), ensuring that the final set of tweets all contained tobacco-related terms (Fig. 1). For example, while “e-cigarette” and related terms (“vape”, “e-cig”, “ecig”) were not seen in the industry’s top hashtags, they were still frequently used in public tweets that contain any keywords in Q1. By combining the search results from Q1 and Q2, 842,754 tweets (final set) were found after duplicates were removed. In other words, each of the resulting tweets in the final set contains any keywords in Q1 and any keywords in Q2.

### Data analysis

To systematically identify the main themes within the collection of 842,754 tweets, we used topic modeling. We specifically chose Latent Dirichlet Allocation (LDA), a widely used unsupervised probabilistic model that discovers latent topics in a corpus by assuming that each document is a mixture of topics and each topic is a mixture of words [20]. LDA is particular well-suited for an exploratory study of a large social media dataset like ours, because it effectively uncovers underlying thematic structures without requiring pre-labeled data. While other methods such as Non-negative Matrix Factorization (NMF) or newer transformer-based models exist, LDA remains a robust and highly interpretable choice for this type of analysis. We selected the 6-topic model as it resulted in the highest coherence score among the number of topics ranging from 3 to 20. We reviewed a random selection of 100 tweets from each topic and named each topic based on the main themes present in the sample tweets (see Table 1 for the size and name of each topic). The 600 tweets served as our initial sample (S1) that all three researchers reviewed in the qualitative analysis to identify key components of the framework.

**Table 1:** Results from topic modeling

Topic name (short name)	Number of tweets	Top keywords	Examples of representative tweets (Redacted)
Vaping to Quit (Cessation)	260,601	‘vaping smoking like cigarette vape smoke year quit people’	“@xxx @xxx @xxx Some people can’t quit smoking cigarettes, but vaping shouldn’t be banned because of a few bad experiences. That’s selfish. I vape, quit smoking after decades, and started again while caregiving for my mom with Alzheimer’s. Vaping is better for me. Let it be!”
Sales and promotion of e-cigarettes and accessories (marketing)	162,341	‘vape vaping vapelife vapefam eliquid ecig ejuice vapeshop vapecommunity’	“Our organic CBD tinctures help relax muscles and ease anxiety. Discover more about Polaris THC-free organic tinctures and find the perfect flavor for your lifestyle @ [URL]”
Against tobacco control policies, distrust of institutions (policy)	161,198	‘vaping tobacco product smoker teen ban vape harm nicotine’	RT @xxx: Interesting. As usual with vaping, follow the money. While youth vaping rates surged, a powerful state lawmaker delayed anti-vaping laws and received nearly $20,000 in campaign contributions from tobacco companies and lobbyists.[URL]
Population level evidence (sciences)	155,928	‘vaping smoking science rt health nicotine tobacco thc cigarette’	“Read this article for more highlights from the e-cigarette summit, especially expert opinions on why #vaping is healthier than smoking! [URL]”
Flavors (flavors)	69,430	‘flavor vape cbd disposable flavour device edible new juice nicotine’	“E-liquid stores are frustrating. Watermelon menthol and menthol tobacco taste like potpourri. I tried mixing watermelon menthol with Juul menthol tobacco, but it still tasted bad. Plus, they’re selling smaller nicotine amounts. Maybe I should just stick to smoking.”
Industry news (industry)	33,256	‘vaping new vape juul different tax news live giveaway country’	“@xxx The extensive PMTA process is stifling American innovation, while China seems to be keeping pace. #wevapewevote”

The qualitative analysis involved three steps. First, we established our criteria for identifying claims inconsistent with scientific consensus by referring to guidelines from major public health bodies, primarily the U.S. Centers for Disease Control and Prevention (CDC) and the World Health Organization (WHO) [21–25]. We selected these organizations not to designate them as sole arbiters of truth, but because they provide the foundational guidance for U.S. and global public health policy and communication. Our objective was to identify claims inconsistent with this prevailing public health messaging. Crucially, we were aware of the contentious nature of some topics, such as the efficacy of e-cigarettes for smoking cessation where scientific consensus is still evolving and views differ globally. To account for this, we adopted a conservative approach: claims arguing for the relative safety of vaping compared to cigarettes were not classified as misinformation. However, we categorized claims of absolute safety (e.g., “vaping is totally safe”), over-generalization without acknowledging risks to certain population (e.g., “vaping is a better choice for everyone who wanted to quit”), and exaggeration (e.g. “vaping is the best alternative to smoking”) as misinformation, as such statements are not supported by established scientific bodies. Following this, we curated a list of additional misleading claims related to different aspects of tobacco products from the literature, including the addictiveness of nicotine, nicotine’s therapeutic functions, e-cigarette/smoking and COVID-19 association, and tobacco company self-promotion [8, 9, 16, 21]. These were used as our “seed” claims [22]. We built our selection criteria by consulting experts and referring to guidelines from established health authorities [23–27]. We also referred to a recently published scoping review of health misinformation to define key components of misleading claims, such as being false, inaccurate, or incorrect, with or without harm intention, and having possible misleading effects [28].

Next, we reviewed each tweet in S1, identifying concepts that matched those mentioned in our seed claims. We then compared the main claim in the tweet with our guideline references and its similarity to the seed claims to determine if that the tweet should be categorized as misleading. We also noted concepts that were not in the seed claims and created new categories, iteratively building the framework.

Here is a redacted example.

*RT @xxx: @xxx*,* @xxx*,* @xxx Did you know that 97% of people use vaping devices as a way to quit a dangerous habit? Vaping is considered the safest alternative to smoking traditional cigarettes. Let’s educate young people to make wise choices and avoid smoking! In my opinion*,* compassion and understanding of this topic are essential*,* which is why it’s clear that no one actually ‘smokes’ an e-cigarette.*

The key concepts we identified in this tweet were smoking cessation and the safety of vaping. Compared with the guidelines, the claim “97% of people use vaping devices as a way to quit a dangerous habit” was not supported because neither the CDC and WHO have affirmed the number or percentage of e-cigarette users who intend use for smoking cessation, or the effectiveness of vaping as a cessation tool.

After reviewing all tweets in the sample, researchers deliberated on their selections, through which we finalized a few selection standards, particularly on how tweets expressing personal experiences should be categorized, and how to categorize tweets that were addressing controversial topics.

When identifying misinformation, if a tweet was mainly an expression of personal opinion or testimonies, it did not necessarily qualify as misinformation. However, if that personal testimony or opinion implied generalizability, it was classified as misinformation. In this example, the phase “vaping is considered the safest alternative” would be identified as misinformation both because it is not accurate, (as CDC and WHO endorse approved pharmacotherapy or complete tobacco cessation as safer than use of e-cigarettes), and the phrase is presented as a generalizable true statement.

The topic of smoking cessation included a mixture of personal experiences and general discussions about the benefits of e-cigarettes as a cessation tool. We categorized all tweets overgeneralizing vaping as an effective cessation tool as misinformation, following the WHO’s guidelines indicating insufficient evidence of the effectiveness of e-cigarette use for cessation at the population level. For instance, if someone tweeted about successfully quitting smoking with vaping, or stated ‘I think vaping is a more effective cessation tool than other methods,’ this was not classified as misinformation because it reflects a personal experience that could be true for that individual. In contrast, a tweet claiming, “I used e-cigarettes to quit smoking, and everyone should have access to vaping, the best cessation tool ever” would be classified as misinformation because it generalizes the effectiveness of vaping as cessation tool, a claim insufficiently supported by scientific evidence.

Similarly, we considered the nuances in claims for topics where there is scientific controversy or contentious discussion, such as the safety of vaping. We did not consider tweets arguing for the relative safety of vaping compared to cigarettes as misinformation, as this claim has gained endorsements from some scientific consensus groups, although these groups may have different views about whether e-cigarettes should be used as a cessation tool [29, 30]. However, any claim using absolute terms to discuss a nuanced topic like the safety and health of vaping was classified as misinformation. For example, a claim stating “vaping is totally safe” or “vaping is the best alternative to smoking” would be classified as misinformation.

To explore the potential for automating the labor-intensive process of misinformation detection, we employed the generative AI model ChatGPT 4.0. The goal of this step was not to replace human analysis but to assess the current capabilities of a state-of-the-art LLM (Large Language Model) in identifying nuanced misinformation claims. ChatGPT 4.0 was selected for this exploratory purpose due to its advanced natural language understanding and wide accessibility at the time the research was conducted. By comparing the AI’s output with our human-coded results, we could better understand the types of misinformation that automated systems can and cannot easily detect, thus highlighting areas where a detailed analytical framework is most needed. To maintain this goal of assessing baseline performance, we did not engage in iterative conversational refinement, such as asking the AI for clarification on its terminology.

We began the AI-assisted analysis by prompting ChatGPT to provide its standard for selecting tobacco-related misinformation as if it were a public health expert in tobacco control. We prompted AI to suppose it was a tobacco control expert, and based on its current knowledge, list the criteria it would use to identify misinformation related to tobacco products.

The following response was generated:

- *Claim that vaping or e-cigarette use is completely safe or significantly safer than traditional tobacco use without acknowledging the existing health risks;*
- *Promote tobacco products as a healthy alternative to smoking;*
- *Suggest that tobacco products have health benefits;*
- *Target younger audiences or non-smokers*,* encouraging them to start using tobacco products;*
- *Misrepresent the addictive nature of nicotine or tobacco.*

Compared with the human-generated standard, the AI standard did not mention cessation but focused on a more general “benefit”. The AI standards also used the term “tobacco products” rather than “e-cigarettes” or “vaping” in several statements, leaving uncertainty about whether the term “tobacco products” would include or exclude e-cigarettes. In general, the statements were very close to what we generally relied on for misinformation classification standards. It was not clear how the AI would classify a nuanced statement or take into account personal opinion as explained above.

We then asked AI to apply these standards and the seed claims to examine the 600-tweet sample and make selections and define categories of misinformation. We prompted AI to read tweets we provided in this sample and compare them with the claims in the attached file (containing a list of sample statements that are not true), and apply the selection criteria it provided previously to identify tweets containing misinformation.

The results were then validated by human researchers, and the tweets that were identified by AI and judged to correctly identify misinformation were incorporated in the final results.

**Fig. 2:**
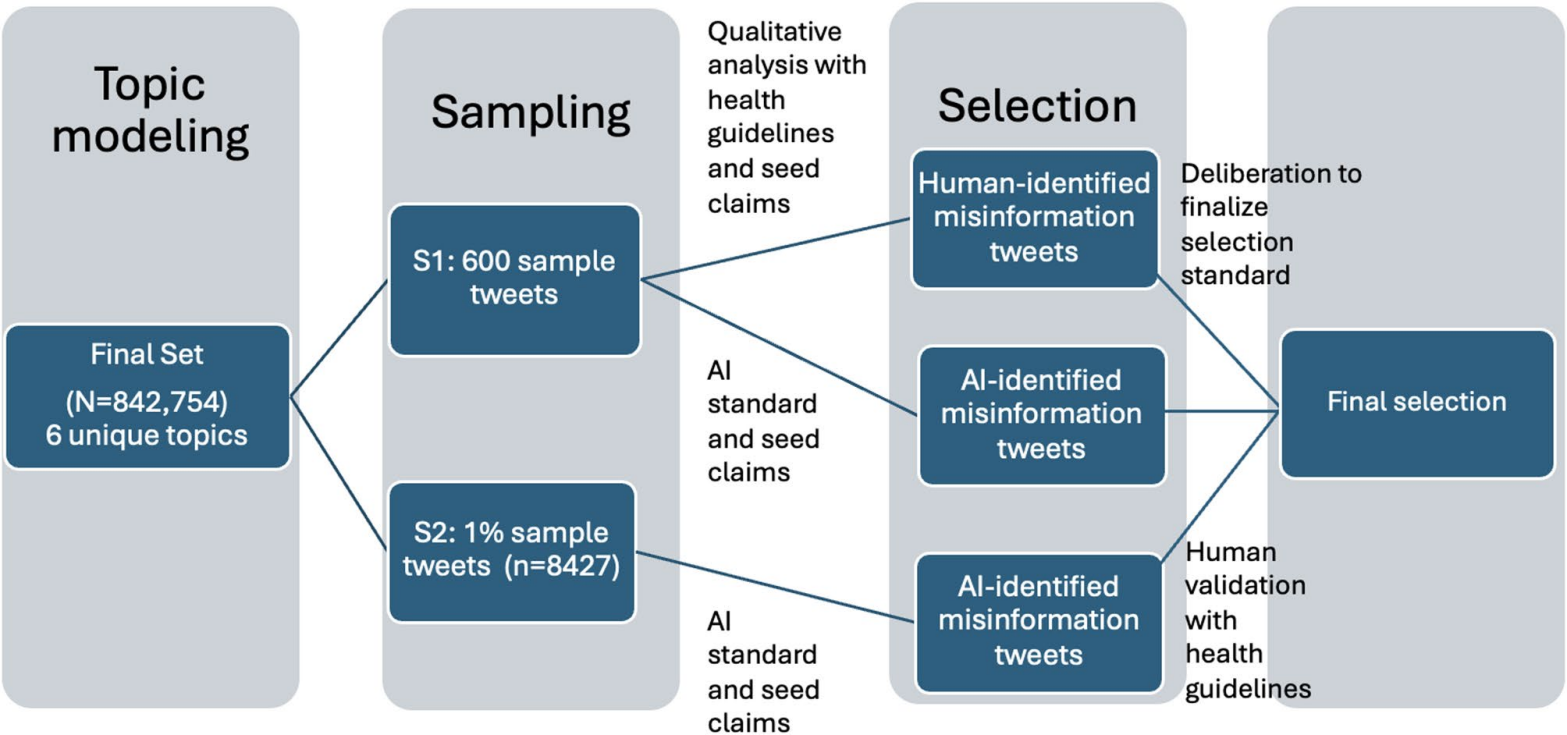
Data analysis process

Next, in order to test the ability of ChatGPT to help automate the misinformation selection and categorization process, we created a larger sample (S2, 1% of the dataset, *N* = 8427), proportional to the sizes of each of the six topics from the topic modeling. Reading the sample tweets and seed claims, it added two standards related to more recent events: (1) nicotine’s protective effects on COVID-19, and (2) e-cigarette or vaping use-associated lung injury (EVALI) is unrelated to vaping. It then produced a few categories that it considered as misinformation. These two additional standards could be resulted from seeing the presence of tweets discussing COVID-19 and EVALI. Finally, we asked ChatGPT to provide its own selection of tweets containing misinformation from the sample, and then validated its selection with human researchers. The validated tweets were also included in the final selection (Fig. 2).

## Results

Six unique topics resulted from topic modeling. These topics informed us the main themes of tweets in this large dataset. The topic names, topic sizes (number of tweets), and redacted examples are listed in Table 1.

Out of the 600 sample tweets in S1, human coders identified 58 as containing misinformation, while the AI identified 21 tweets as misinformation, eight [8] of which were also found in the human-identified 58 tweets, all containing pro-vaping content. The other 13 AI-identified tweets did not meet the health guidelines and researcher agreements and were thus excluded from the final selection. For the additional 8427 tweet-sample (S2), 557 tweets were identified by AI, 121 of which were validated by human coders applying the official health guidelines. Our final selection of tweets containing misinformation was 169, including the 58 human identified tweets in S1 and the 121 human validated AI-identified tweets.

**Fig. 3:**
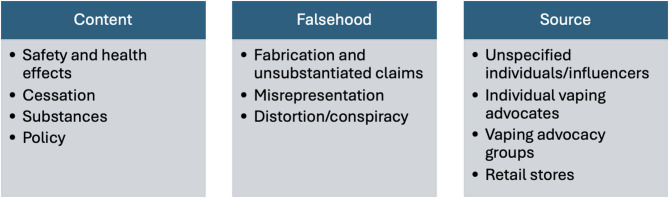
The tobacco-related information framework

Through a qualitative analysis of the entire sample of 169 human-validated misleading tweets, we identified three key components (dimensions) for categorization:1) content, 2) type of falsehood, and 3) source. For each component (dimension), we also defined several sub-categories, as shown in Fig. 3.

### Content

The content dimension was mainly informed by the topic modeling results (Table 1) and the categories provided by AI. Misinformation categories provided and named by AI include: Nicotine reduces COVID-19 risks, vaping is completely safe and risk free, EVALI is unrelated to vaping, nicotine is not or slightly addictive, economic and regulatory influences (meaning the impact of strict policies like “vape ban” on the economy, by destroying small businesses manufacturing e-cigarettes and letting Big Tobacco produce more cigarettes to kill people), and vaping is an effective cessation tool for everyone. Four sub-categories were summarized: safety and health effects, cessation, substances, and policy. (Table 2)

**Table 2:** Three dimensions and examples

Dimension	Sub-category	Example
Content	Safety and health effects	*@xxx Popcorn lung is caused by the chemical diacetyl*,* which isn’t present in most vape juices. As long as you research the juices you buy and avoid sketchy sources*,* you’re fine. Plus*,* vaping nicotine has been around since the 60s.*
	Cessation	Vaping is the simplest method to quit! It’s 95% safer than smoking! #EndVapeBan #QuitSmoking #StopTheLies #VapingSavesLives*
	Substance	*RT @xxxx Nothing is 100% safe*,* and it never will be! Even aspirin can cause stomach bleeding as a side effect. Nicotine vaping is at least 95% safer*,* closer to 97–98% now*,* than smoking.*
	Policy	*“@xxx This is why the #WHO #FCTC fights against vaping*,* which helps people quit smoking and reduces cancer risk. Tobacco and cancer industries profit $$$. [URL]”*
Falseness of Claim	Misrepresentation	*@xxx @xxx excellent news! all people predisposed to #nicotine use should have access to the #harmless and effective #vaping alternative to cigarettes regardless of age.*
	Distortion	*I hope when they ban all vaping devices we aren’t weak and go back to big tobacco like the government is trying to make us do*
	Unsubstantiated claim	*i realized today that i have no time for seeing people vape! people don’t know how bad it is and what it can do to you! what does vaping do? the answe[r] is it caused depression and anxiety!*
Source	Vaping advocacy group	*RT @xxx: @xxx*,* @xxx*,* @xxx Did you know that 97% of people use vaping devices as a way to quit a dangerous habit? Vaping is considered the safest alternative to smoking traditional cigarettes. Let’s educate young people to make wise choices and avoid smoking! In my opinion*,* compassion and understanding of this topic are essential*,* which is why it’s clear that no one actually ‘smokes’ an e-cigarette*
	Individual vaping advocate	Individuals who have claimed certain areas of expertise and market products, with paid or unpaid promotions of certain products
	Unspecified individual and influencer	*I realized today that I have no time for seeing people vape! people don’t know how bad it is and what it can do to you! what does vaping do? the answer[r] is it caused depression and anxiety!*
	Retail store	*the cdc is to blame for more americans than ever being misinformed about vaping and e-cigarettes [URL] via @xxx*

*While endorsed by established and reputable health institutions like Public Health England [41], this claim has often been used out of context to show that vaping is always safer than cigarettes and could be misleading. Researchers have used this statement to test misinformation perception [12]

#### Safety and health effects

This category refers to claims about health benefits of products, mostly e-cigarettes, usually about how the product is the safest alternative to smoking, and the denial of association between vaping and some health outcomes, such as EVALI.

#### Cessation

This category mainly contains the overstated claims that e-cigarettes are regarded as the best way of helping people quit smoking.

#### Substances

This category includes tweets that discuss the different types of substances in the devices, such as comparing the health effects of cannabis to tobacco, or the addictiveness of nicotine.

#### Policy

This category includes discussions about tobacco control policies and their possible impacts, for example, the argument that a “vape ban” could hurt small businesses while benefit Big Tobacco, and also that the government is helping Big Tobacco to kill more people.

Among the 169 selected tweets, 79 were about safety and health effects (mainly about vaping), making it the largest misinformation topic. Policy was also a common topic (49 tweets).

### Types of falsehood

The falsehood of the claims refers to the degree to which they deviate from reality or existing scientific consensus. Drawing on existing typology [14], we identified three types of falsehood: (a) fabrication and unsubstantiated claims, (b) misrepresentation, and (c) distortion/conspiracy. Among the 169 tweets, 30 had fabrication and unsubstantiated claims, 122 had misrepresentation, and 44 had distortion/conspiracy.

#### Fabrication and unsubstantiated claims

Fabrication and unsubstantiated claims include those with factual errors or those lacking scientific evidence. For example, the claim “Vaping reduces the risks of COVID-19” is a fabrication, as there is no scientific evidence to support it.

#### Misrepresentation

Misrepresentation involves claims that may be true or partially true but are inaccurately presented or interpreted. These claims can be misused (cited out of context), exaggerated (overstated), or employed with inappropriate analogies, leading to misleading conclusions. In our analysis, 36 of the 58 human-identified tweets fell into this category.

For instance, the claim “Vaping is completely safe and risk-free” is an exaggeration. Similarly, “Vaping is an effective cessation tool for me, so it should work for everyone” overstates its benefits, neglecting the specific conditions under which vaping can be an effective cessation tool, such as adult use only, complete switching from cigarettes, and the fact that it is never safe for youth or non-smokers [23]. A notable pattern in our analysis was the misinterpretation of policies.

Consider the tweet: “Are you aware that vaping doesn’t contain any of the numerous carcinogenic chemicals found in traditional cigarettes that are approved by the FDA in every cigarette? Make the switch to vaping and avoid breathing in the harmful compounds that have FDA approval.” This tweet misinterprets the “FDA approval of cigarette products,” mistakenly equating the FDA’s marketing authorization with an endorsement of the product’s safety [25]. 

Another example is the frequent reference to “vape ban” policy. Such claims are exaggerated, as policies do not completely ban the manufacturing and sales of e-cigarettes but rather restrict their reach to youth or ban the sales of flavored products [31]. 

#### Distortion/conspiracy

Distortion/conspiracy claims present a distorted reality and skepticism of established institutions such as the government, businesses, NGOs, and scientific community. These claims typically involve conspiracy narratives about policies, suggesting hidden plots by powerful entities benefiting from a policy at the expense of individual freedom or the general public. A typical example is the claim: “The government bans vape to protect the profits of Big Tobacco/Pharma and can financially benefit from it, at the expenses of small businesses,” or “This is why the #WHO #FCTC fights against vaping, which helps people quit smoking and lowers cancer risk. Tobacco and cancer industries profit $$$.”

### Sources

#### Individual vaping advocates

Finally, we examined the sources likely to spread misinformation. Analysis of the user profiles of the selected tweets showed that individual vaping advocates constituted the largest category of sources, responsible for 79 out of the 169 misinformation tweets. Further investigation of their accounts revealed that these users often had a history of posting pro-vaping content, and many had bios or usernames related to the pro-vaping mission.

#### Unspecified individuals/influencers

Another misinformation-spreading source was unspecified individuals or influencers. They are not posting specifically about tobacco-related topics, and 45 out of the 169 tweets were created by them.

#### Vaping advocacy groups

Unlike individual advocates, the vaping advocacy groups are the ones that are known for their advocacy agenda in favor of industry interests, sometimes with verified accounts. 12 out of the 169 tweets were posted by this category.

#### Retail stores

This category refers to online accounts that are selling vaping or cannabis products. 12 of the 169 tweets were posted by them.

Additionally, 31 original tweets were missing as of the time of analysis, and the sources could not be identified.

While our data collection started with industry tweets, we have not seen misinformation tweets that came from the tobacco industry or about the industry in our analytical sample. Instead, in the pro-vaping advocacy tweets, the “Big Tobacco” was seen to be working with the government in the anti-vaping policies, as these policies were seen to be serving their interests to destroy small businesses and individual freedom.

## Discussion

This examination of Twitter data informed the development of an analytical framework for tobacco-related information with three core components: content, type of falsehood, and source. Our framework integrates multiple elements of tobacco-related misinformation. Systematically developing a tobacco-related information framework from social media content with the assistance of generative AI revealed several important characteristics of tobacco-related information and the challenges in identification and categorization.

First, we observed that tobacco-related misinformation often manifests subtly compared with other areas like politics where fabrication and engineered falsehoods are more common [32]. Our sample did not see many completely unsupported claims, totally fabricated stories, or “explicit misinformation” [33]. In most cases, misinformation typically involved overstated safety or risks, misinterpretation of policies, or misuse of scientific or expert consensus, which has been characterized as “implicit misinformation” [33]. The claims are misleading and ambiguous rather than totally deceptive. This also means that in most cases, the falseness of a tobacco-related claim may not be readily apparent to a non-expert audience.

These nuances in the content may have affected the results of AI selection. As the results showed, only about 1% of the tweets in the sample of 600 tweets (S1) were identified by both human researchers and AI as misinformation, and for S2 (8427 tweets), only 21.7% of the AI-identified tweets (121 out of 557) were valid when assessed by human coders. Most of those tweets had extreme terms and included a distortion of reality or conspiracy attacking governmental agencies or other reputable institutions. AI may have recognized these tweets as misinformation because of the presence of extreme terms, but AI had more difficulty accurately identifying tweets with relative safety claims.

Compared with human categorization, the AI categories were one dimensional, focusing on content, and did not differentiate the types of falsehood in those claims or consider sources because this dataset does not have user information directly accessible. AI also mistakenly identified tweets expressing relative safety of vaping as misinformation, possibly due to the tendency to classify the presence of any “safe” or “healthy” terms as misinformation.

Another revealing limitation was the AI’s imprecise terminology. The selection criteria provided by AI used the general term “tobacco products” rather than the more specific “e-cigarettes” or “vaping”. This is a critical distinction in modern tobacco control. This imprecision suggests that the model, despite its vast training, may lock the domain-specific knowledge to differentiate between product categories that are essentially central to current policy and health debates. Our study highlights a key risk in using generative AI for specialized domains: an inability to grasp context-specific nuance that is readily apparent to human expert.

The contentious nature of the discussions and lack of scientific consensus related to new tobacco products made it challenging to categorize claims as misleading. The analysis of tweets showed that opinions around the safety and risks of e-cigarettes were highly contentious. While neither established guidelines from health authorities like WHO and CDC support e-cigarettes as an effective cessation tool, other health authorities such as the Royal College of Physicians have endorsed e-cigarettes as significantly less harmful and an effective cessation method [30]. This divergence in views makes categorizing and fact-checking difficult and can introduce bias. As these products are relatively new to the market and research may not be able to keep up with the rapid evolution of the tobacco marketplace [6], the lack of up-to-date evidence also contributes to this challenge.

The subtlety of tobacco misinformation also makes equitable information access more difficult. It is more challenging for people with lower levels of information literacy and lack of access to credible sources to identify the subtlety of falseness in claims, making them more susceptible to be misled [2]. Researchers, and those designing health campaigns, and interventions should consider addressing ambiguity in messages, and elements in addition to content issues, such as conspiracy theories or source credibility, that may help to identify claims.

Second, our findings suggested that misconceptions about policy and regulation are significant misinformation topics, similar to previous research about other health topics that made a distinction between professional (health-related) and political (policy-related) misinformation [34]. In our framework, policy was a distinctive misinformation topic with many tweets misinterpreting policies. Such misinterpretations are often part of a distorted view of established authorities, which are seen as serving the interests of the tobacco industry by “banning” vapes, reflecting an overall distrust of institutions [8] that could hinder policies benefiting public health.

The common presence of policy misinformation in the realm of tobacco also suggests that tobacco-related misinformation aligns with a larger network of conspiracy theories in other health topics, such as fluoride [34], vaccine [35], and 5G and COVID-19 [36]. These patterned claims with a wide range of variety of topics showed that rather than considering conspiracy theories as a content or theme category as it has been classified in prior studies [14, 37, 38], it should be a category of falsehood to highlight how this kind of message presents distorted views of reality and makes artificial connections between unrelated facts to foster anti-science and anti-institution agendas.

Additionally, our analysis highlighted that the most common source of tobacco-related misinformation was pro-vaping individuals who did not assume an influencer role, distinct from social bots [39], pro-vaping advocacy groups [10] and influencers [40], which have been previously identified as the misinformation sources in the literature. These findings underscore the need for further investigation, possibly through network analysis, to assess and characterize industry and advocacy group connections to these individuals. Among other sources, pro-vaping advocacy groups on Twitter were known for their presence and the role of “gatekeeping” in spreading misinformation [10]. Retailers could promote products with unsubstantiated health or safety claims that were not authorized for marketing [25], and their roles in spreading misinformation should be further examined.

The development of our framework was supported by a combination of machine learning and human coding to discern subtle nuances in social media posts, along with the use of generative AI for selection and categorization. Although generative AI showed promise in organizing information and aligning with established references, we noted limitations in its keyword matching approach, suggesting the need for iterative prompt adjustments for improved accuracy and more advanced NLP techniques and to explore strategies.

This early-stage exploration, which serves as a first step for formal taxonomy building [15], revealed the complexity of categorizing claims due to contentious discussions and a lack of scientific consensus, particularly concerning new tobacco products like e-cigarettes. Future research should refine and validate our framework by incorporating other media sources, expert opinions [15], and confirmed misinformation content. Future research should also address enhancing the accuracy and utility of AI tools in misinformation detection and categorization.

### Limitations

This framework is based solely on Twitter’s textual content and is thus subject to limitations. It has not been validated for multimedia (e.g., images, videos) or for content from other platforms. Additionally, the influence of social bots and the network dynamics of message spread were outside this study’s scope. While future research is needed to extend the framework to these contexts, this study provides a foundational taxonomy for textual tobacco-related misinformation.

Another limitation is data availability and representativeness. This study was limited to tweets from 2020 to 2022. As Twitter became X and the API policy changed, free access for research purpose is no longer possible and not currently a viable solution for collecting more recent data. Additionally, the dataset may be incomplete, as some original tweets may have been removed by the platform. We also recognize that tweets do not represent all opinions on these topics. They only represent opinions shared by the platform’s users on a publicly available social media platform, which does not reflect the opinions in the entire population.

Finally, the exploratory nature of the AI-assisted analysis has its own limitations. The lack of agreement between AI and human results shows that the AI approach struggled to detect the nuances in more complex claims, even though it could help flag posts containing extreme terms of conspiracy narratives. This highlights the challenges that current automated system face in this area.

This preliminary analytical framework needs further development and validation, which could eventually enhance the ability of computer-assisted models or AI to effectively identify tobacco-related misinformation.

## Conclusion

This study introduces an analytical framework for categorizing tobacco-related misinformation on social media developed through a combination of human expertise and AI-assisted analysis.

It highlights the subtle nature of tobacco-related misinformation, which poses significant challenges for non-expert users and individuals with limited information literacy. Given the subtlety and complexity of misinformation on social media, there is a critical need for public health initiatives to communicate clear and precise scientific information to debunk misleading claims. Targeted educational programs should consider these nuances of misinformation to craft tailored messages to guide people, particularly those with lower information literacy and higher rates of tobacco use, on how to access credible sources and effectively utilize information resources. The insights gained from this study can guide public health policymakers and regulatory agencies in effective public communications about new policies and regulatory decisions, taking into account potential misinterpretations and the pervasive spread of misinformation on social media. For example, it may facilitate health communicators to be more proactive in anticipating potential types misinformation and prepare FAQs, evidence-based info-graphics or other media in advance of releasing new policies.

## Data Availability

The datasets generated and analyzed during the current study are not publicly available due to the presence of personal identifiable information, but the de-identified datasets are available from the corresponding author on reasonable request.

## Abbreviations

AI: Artificial Intelligence
API: Application programming interface
CDC: U.S. Centers for Disease Control and Prevention
EVALI: E-cigarette or vaping use-associated lung injury
FDA: Food and Drug Administration
LDA: Latent Dirichlet Allocation
LLM: Large Language Model
NLP: Natural Language Processing
NMF: Non-negative Matrix Factorization
WHO: World Health Organization

## References

[1] Tan ASL, Bigman CA, Misinformation About Commercial Tobacco Products on Social Media—Implications and Research Opportunities for Reducing Tobacco-Related Health Disparities. Am J Public Health. 2020 Oct;110(S3):S281–3. Available from: https://ajph.aphapublications.org/doi/full/10.2105/AJPH.2020.305910. cited 11 Oct 2021. 10.2105/AJPH.2020.305910 PMC7532322 33001728

[2] Seo H, Blomberg M, Altschwager D, Vu HT. Vulnerable populations and misinformation: a mixed-methods approach to underserved older adults’ online information assessment. New Media Soc. 2021;23(7):2012–33. 10.1177/1461444820925041.

[3] Harlow AF, Stokes A, Brooks DR. Socioeconomic and Racial/Ethnic Differences in E-Cigarette Uptake Among Cigarette Smokers: Longitudinal Analysis of the Population Assessment of Tobacco and Health (PATH) Study. Nicotine & Tobacco Research. 2019 Sep 19;21(10):1385–93. Available from: https://academic.oup.com/ntr/article/21/10/1385/5050238. cited 17 Apr 2024. 10.1093/ntr/nty141 PMC6751515 29986109

[4] Viswanath K, Nagler RH, Bigman-Galimore CA, McCauley MP, Jung M, Ramanadhan S. The Communications Revolution and Health Inequalities in the 21st Century: Implications for Cancer Control. Cancer Epidemiology, Biomarkers & Prevention. 2012 Oct 1;21(10):1701–8. Available from: https://aacrjournals.org/cebp/article/21/10/1701/68964/The-Communications-Revolution-and-Health. cited 16 Apr 2024. 10.1158/1055-9965.EPI-12-0852 PMC3468900 23045545

[5] Suarez-Lledo V, Alvarez-Galvez J. Prevalence of Health Misinformation on Social Media: Systematic Review. Journal of Medical Internet Research. 2021;23(1). Available from:://WOS:000609010900004. 10.2196/17187 PMC7857950 33470931

[6] Ling PM, Kim M, Egbe CO, Patanavanich R, Pinho M, Hendlin Y. Moving targets: how the rapidly changing tobacco and nicotine landscape creates advertising and promotion policy challenges. Tob Control. 2022;31(2):222–8. 10.1136/tobaccocontrol-2021-056552. 35241592 10.1136/tobaccocontrol-2021-056552 PMC9233523

[7] Albarracin D, Romer D, Jones C, Hall Jamieson K, Jamieson P. Misleading claims about tobacco products in YouTube videos: experimental effects of misinformation on unhealthy attitudes. J Med Internet Res. 2018;20(6):10. 10.2196/jmir.9959. 10.2196/jmir.9959 PMC6045787 29959113

[8] Al-Rawi A, Blackwell B, Zemenchik K, Lee K. Twitter misinformation discourses about vaping: systematic content analysis. J Med Internet Res. 2023;25:e49416. 37948118 10.2196/49416 PMC10674139

[9] Sidani J, Hoffman B, Colditz J, Melcher E, Taneja S, Shensa A, et al. E-cigarette-related nicotine misinformation on social media. Subst Use Misuse. 2022;57(4):588–94. 35068338 10.1080/10826084.2022.2026963 PMC9257904

[10] Silver N, Kierstead E, Kostygina G, Tran H, Briggs J, Emery S et al. The Influence of Provaping Gatewatchers on the Dissemination of COVID-19 Misinformation on Twitter: Analysis of Twitter Discourse Regarding Nicotine and the COVID-19 Pandemic. J Med Internet Res. 2022 Sep 22;24(9):e40331. Available from: https://www.jmir.org/2022/9/e40331. cited 23 Jul 2023. 10.2196/40331 PMC9506503 36070451

[11] Silver N, Kierstead E, Tran B, Sparrock L, Vallone D, Schillo B. Belief and recall of nicotine as therapeutic for COVID-19 may undermine e-cigarette quitting behavior. Health Educ Behav. 2022;109019812211091. 10.1177/10901981221109127. 10.1177/10901981221109127 35848331

[12] Wang W, Huang Y. Countering the harmless E-cigarette myth: the interplay of message format, message sidedness, and prior experience with E-cigarette use in misinformation correction. Sci Commun. 2021;43(2):170–98.

[13] Li K, Shin D. Correcting E-Cigarette Misinformation on Social Media: Responses from UAE Nationals Who Smoke. J Broadcast Electron Media. 2023;67(3):376–96. 10.1080/08838151.2023.2201506.

[14] Kata A. A postmodern Pandora’s box: Anti-vaccination misinformation on the Internet. Vaccine. 2010 Feb;28(7):1709–16. Available from: https://linkinghub.elsevier.com/retrieve/pii/S0264410X09019264. cited 25 May 2020. 10.1016/j.vaccine.2009.12.022 20045099

[15] Stureborg R, Nichols J, Dhingra B, Yang J, Orenstein W, Bednarczyk RA et al. Development and validation of VaxConcerns: A taxonomy of vaccine concerns and misinformation with Crowdsource-Viability. Vaccine. 2024 Mar;S0264410X2400255X. Available from: https://linkinghub.elsevier.com/retrieve/pii/S0264410X2400255X. cited 25 Mar 2024. 10.1016/j.vaccine.2024.02.081 38521676

[16] Liang Y, Zheng X, Zeng DD, Zhou X, Leischow SJ, Chung W. Exploring How the Tobacco Industry Presents and Promotes Itself in Social Media. J Med Internet Res. 2015 Jan 21;17(1):e24. Available from: http://www.jmir.org/2015/1/e24/. cited 17 Nov 2021. 10.2196/jmir.3665 PMC4319084 25608524

[17] Watts C, Hefler M, Freeman B. ‘We have a rich heritage and, we believe, a bright future’: how transnational tobacco companies are using Twitter to oppose policy and shape their public identity. Tob Control. 2019 Mar;28(2):227–32. Available from: https://tobaccocontrol.bmj.com/lookup/doi/10.1136/tobaccocontrol-2017-054188. cited 16 May 2022. 10.1136/tobaccocontrol-2017-054188 29666168

[18] Valdez D, Unger JB. Difficulty Regulating Social Media Content of Age-Restricted Products: Comparing JUUL’s Official Twitter Timeline and Social Media Content About JUUL. JMIR Infodemiology. 2021 Dec 7;1(1):e29011. Available from: https://infodemiology.jmir.org/2021/1/e29011. cited 11 Dec 2021. 10.2196/29011 PMC10014088 37114198

[19] Allem JP, Rodriguez V, Pattarroyo M, Ramirez CM, Beard TA, Soto D et al. Spanish-Language Tobacco-Related Posts on Twitter: Content Analysis. Nicotine and Tobacco Research. 2024 May 22;26(6):759–63. Available from: https://academic.oup.com/ntr/article/26/6/759/7371636. cited 5 Jun 2024. 10.1093/ntr/ntad220 PMC11519019 37942524

[20] Blei DM, Ng AY, Jordan MI. Latent Dirichlet Allocation. The Journal of Machine Learning Research. 2023;3:993–1022. Available from: https://dl.acm.org/doi/10.5555/944919.944937.

[21] Sidani J, Hoffman B, Colditz J, Wolynn R, Hsiao L, Chu K, et al. Discussions and misinformation about electronic nicotine delivery systems and COVID-19: qualitative analysis of Twitter content. JMIR Form Res. 2022. 10.2196/26335. 35311684 10.2196/26335 PMC9009382

[22] ElSherief M, Sumner S, Krishnasamy V, Jones C, Law R, Kacha-Ochana A et al. Identification of Myths and Misinformation About Treatment for Opioid Use Disorder on Social Media: Infodemiology Study. JMIR Form Res. 2024 Feb 23;8:e44726. Available from: https://formative.jmir.org/2024/1/e44726. cited 26 Feb 2024. 10.2196/44726 PMC10924265 38393772

[23] CDC, Smoking. and Tobacco Use. 2024. E-Cigarettes (Vapes). Available from: https://www.cdc.gov/tobacco/e-cigarettes/index.html. cited 26 Jun 2024.

[24] CDC, Smoking. and Tobacco Use. 2024. Heated Tobacco Products. Available from: https://www.cdc.gov/tobacco/other-tobacco-products/heated-tobacco-products.html. cited 26 Jun 2024.

[25] FDA. Health Fraud [Internet]. 2023 [cited 2024 Jun 24]. Available from: https://www.fda.gov/tobacco-products/health-effects-tobacco-use/health-fraud

[26] World Health Organization. Tobacco: E-cigarettes [Internet]. [cited 2024 Jun 26]. Available from: https://www.who.int/news-room/questions-and-answers/item/tobacco-e-cigarettes

[27] World Health Organization. Heated tobacco products: information sheet – 2nd edition [Internet]. [cited 2024 Jun 26]. Available from: https://www.who.int/publications/i/item/WHO-HEP-HPR-2020.2

[28] Mikati IKE, Hoteit R, Harb T, Zein OE, Piggott T, Melki J et al. Defining Misinformation and Related Terms in Health-Related Literature: Scoping Review. Journal of Medical Internet Research. 2023 Aug 9;25(1):e45731. Available from: https://www.jmir.org/2023/1/e45731. cited 14 Aug 2023. 10.2196/45731 PMC10414029 37556184

[29] American Cancer Society. American Cancer Society Position Statement on Electronic Cigarettes [Internet]. [cited 2024 Jun 25]. Available from: https://www.cancer.org/cancer/risk-prevention/tobacco/e-cigarettes-vaping/e-cigarette-position-statement.html

[30] Royal College of Physicians. Nicotine without smoke: tobacco harm reduction. London: RCP; 2016 Apr.

[31] FDA. FDA Warns Online Retailers to Stop Selling Illegal E-Cigarettes Popular Among Youth [Internet]. 2023 [cited 2024 Jul 14]. Available from: https://www.fda.gov/apology_objects/abuse-detection-apology.html

[32] Oehmichen A, Hua K, Amador Diaz Lopez J, Molina-Solana M, Gomez-Romero J, Guo Y. ke. Not All Lies Are Equal. A Study Into the Engineering of Political Misinformation in the 2016 US Presidential Election. IEEE Access. 2019;7:126305–14. Available from: https://ieeexplore.ieee.org/document/8819953/. cited 30 May 2024.

[33] Cappella JN, Maloney E, Ophir Y, Brennan E. Interventions to Correct Misinformation about Tobacco Products. tobacco reg sci. 2015;1(2):186–97. 10.18001/TRS.1.2.8 PMC4849128 27135046

[34] Lotto M, Menezes TS, Hussain IZ, Tsao SF, Butt ZA, Morita PP et al. Characterization of False or Misleading Fluoride Content on Instagram: Infodemiology Study. Journal of Medical Internet Research. 2022 May 19;24(5):e37519. Available from: https://www.jmir.org/2022/5/e37519. cited 10 Aug 2023. 10.2196/37519 PMC9164089 35588055

[35] Jamison AM, Broniatowski DA, Dredze M, Wood-Doughty Z, Khan D, Quinn SC. Vaccine-related advertising in the Facebook Ad Archive. Vaccine. 2020 Jan;38(3):512–20. Available from: https://linkinghub.elsevier.com/retrieve/pii/S0264410X1931446X. cited 15 May 2020. 10.1016/j.vaccine.2019.10.066 PMC6954281 31732327

[36] Ahmed W, Vidal-Alaball J, Downing J, López Seguí F. COVID-19 and the 5G Conspiracy Theory: Social Network Analysis of Twitter Data. J Med Internet Res. 2020 May 6;22(5):e19458. Available from: http://www.jmir.org/2020/5/e19458/. cited 26 Jun 2020. 10.2196/19458 PMC7205032 32352383

[37] Jamison AM, Broniatowski DA, Dredze M, Wood-Doughty Z, Khan D, Quinn SC. Vaccine-related advertising in the Facebook ad archive. Vaccine [Internet]. 2020;38(3):512–20. Available from:://WOS:000509816900012. 31732327 10.1016/j.vaccine.2019.10.066 PMC6954281

[38] Schmid P, Altay S, Scherer LD. The Psychological Impacts and Message Features of Health Misinformation: A Systematic Review of Randomized Controlled Trials. European Psychologist. 2023 Jul;28(3):162–72. Available from: https://econtent.hogrefe.com/doi/10.1027/1016-9040/a000494. cited 3 Apr 2024.

[39] Allem JP, Ferrara E, Uppu SP, Cruz TB, Unger JB. E-Cigarette Surveillance With Social Media Data: Social Bots, Emerging Topics, and Trends. JMIR Public Health Surveill. 2017 Dec 20;3(4):e98. Available from: http://publichealth.jmir.org/2017/4/e98/. cited Nov 17 2021. 10.2196/publichealth.8641 PMC5752967 29263018

[40] Zhou R, Xie Z, Tang Q, Li D. Social Network Analysis of e-Cigarette–Related Social Media Influencers on Twitter/X: Observational Study. JMIR Formative Research. 2024 Apr 1;8(1):e53666. Available from: https://formative.jmir.org/2024/1/e53666. cited Apr 5 2024. 10.2196/53666 PMC11019427 38557555

[41] Public Health England. GOV.UK. 2015 [cited 2024 Jul 19]. E-cigarettes around 95% less harmful than tobacco estimates landmark review. Available from: https://www.gov.uk/government/news/e-cigarettes-around-95-less-harmful-than-tobacco-estimates-landmark-review

